# Somatosensory Space *Abridged*: Rapid Change in Tactile Localization Using a Motion Stimulus

**DOI:** 10.1371/journal.pone.0090892

**Published:** 2014-03-06

**Authors:** Tatjana Seizova-Cajic, Janet L. Taylor

**Affiliations:** 1 Neuroscience Research Australia, Sydney, Australia; 2 University of New South Wales, Sydney, Australia; 3 Faculty of Health Sciences, University of Sydney, Sydney, Australia; Birkbeck, University of London, United Kingdom

## Abstract

**Introduction:**

Organization of tactile input into somatotopic maps enables us to localize stimuli on the skin. *Temporal* relationships between stimuli are important in maintaining the maps and influence perceived locations of discrete stimuli. This points to the spatiotemporal stimulation sequences experienced as motion as a potential powerful organizing principle for spatial maps. We ask whether continuity of the motion determines perceived location of areas in the motion path using a novel tactile stimulus designed to ‘convince’ the brain that a patch of skin does not exist by rapidly skipping over it.

**Method:**

Two brushes, fixed 9 cm apart, moved back and forth along the forearm (at 14.5 cm s^−1^), crossing a 10-cm long ‘occluder’, which prevented skin stimulation in the middle of the motion path. Crucially, only one brush contacted the skin at any one time, and the occluder was traversed almost instantaneously. Participants pointed with the other arm towards the felt location of the brush when it was briefly halted during repetitive motion, and also reported where they felt they had been brushed.

**Results:**

Participants did not report the 10-cm gap in stimulation – the motion path was perceptually completed. Pointing results showed that brush path was ‘abridged’: locations immediately on either side of the occluder, as well as location at the ends of the brush path, were perceived to be >3 cm closer to each other than in the control condition (F(1,9) = 7.19; p = .025 and F(1,9) = 6.02, p = .037 respectively). This bias increased with prolonged stimulation.

**Conclusions:**

An illusion of completion induced by our Abridging stimulus is accompanied by gross mislocalization, suggesting that motion determines perceived locations. The effect reveals the operation of Gestalt principles in touch and suggests the existence of dynamic maps that quickly adjust to the current input pattern.

## Introduction

Spatial organization of sensory input is common in the cortex, including the organization of tactile input into somatotopic maps. This organization enables us to localize stimuli on the skin and perform other spatial tasks [1], [2]. The maps are plastic [3], [4], and ‘self-organizing’ [5], [6] i.e., based on the type of learning (also used in artificial networks) in which probabilities present in the input come to be reflected in the neural connections through Hebbian-like rules [3]–[6]. Neurophysiological studies have demonstrated the significance of *temporal* relationships between stimuli. For example, in a classical study on monkeys [7], a flap of skin was relocated to a different finger, while its original innervation was fully preserved. The relocation created new spatial relationships between previously remote skin patches. This naturally brought about a change in the temporal pattern of their inputs, such that the new neighboring locations were now co-stimulated with high probability. The receptive fields of cortical neurons also changed, reflecting the new neighborhood relations, and have thus been described as ‘time-based constructs’ [7]. Findings like these motivated an extension of formal self-organizing models to include temporal stimulus structure [8].

Psychophysical studies also show the importance of stimulus timing in determining perceived spatial relationships. After days of co-stimulation of thumb and little finger, when asked to say which digit is touched with a threshold-level stimulus, blindfolded participants who would normally err mostly by indicating the neighboring digit, now mislocalize closer to the previously co-stimulated digit [9]. Other known effects do not require long prior exposure to the stimulus pattern but occur at once. If three tactile stimuli are presented with equal spatial intervals but unequal temporal intervals, perception of the spatial intervals corresponds to the timing. This phenomenon is known as the Tau effect [10]. In an illusion known as the cutaneous rabbit or sensory saltation, tactile (or visual) stimuli presented in quick succession are ‘drawn’ toward each other and perceived in locations remote from their true positions [11]–[15].

The importance of timing in determining spatial relationships points to the spatiotemporal stimulation sequences experienced as *motion* as a potential powerful organizing principle for spatial maps. Stimulus motion across the receptor surface is the most ubiquitous form of natural stimulation in both touch and vision. The idea that it is *the* means to organize spatial maps dates back at least to the 19^th^ century: “When, in movement of the body, a stimulus changes its region of stimulation, the local signs change, and successive local signs are the things of adjacent localities” [Lotze, 1852 cited in 16, p. 268]. However, systematic empirical investigation of the effects of cutaneous motion on perception of position is lacking.

**Figure 1:**
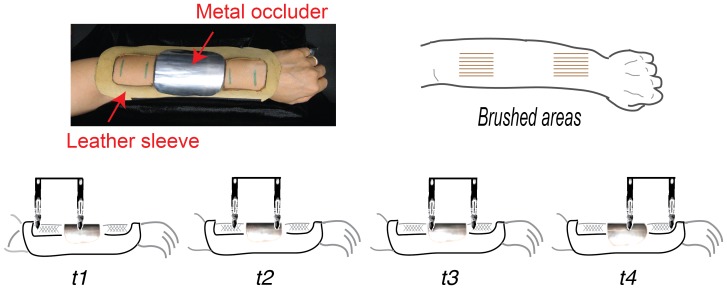
‘Abridging’ stimulus. The stimulus was applied to the forearm using two rigidly connected brushes that moved proximal to distal and distal to proximal. Only one brush touched the skin at any moment because in the middle of the forearm was a metal ‘occluder’ that prevented brush stimulation of the skin underneath. Critically, as the proximal brush climbed onto the occluder, the distal brush descended onto the skin (t2–t3), and continued to move in the same direction (t4), creating a fragmented motion path but almost no interruption in time. Direction was then reversed and the sequence repeated many times (see Method).

Here we studied motion as the organizing principle of spatial maps in conscious healthy humans. We ask whether continuity of the motion determines perceived location of areas in the motion path. To address this question, we developed a novel tactile stimulus, designed to ‘convince’ the brain that a patch of skin does not exist by skipping over it during continuous motion across the skin (see Fig. 1). The stimulus presents the perceiver with two motion fragments that are spatially separated but continuous in time. Its uniform speed and continuing motion in the same direction provide strong cues that a single moving object generates the input [17], [18]. We expected this stimulus to change where people felt they were being touched.

## Method

### Ethics Statement

The study was approved by the University of Sydney and the University of New South Wales ethics committees. Participants provided their written informed consent to participate in the study, according to the procedure approved by the ethics committees.

### Participants

Ten naïve participants completed the experiment (six females), age range from 18 to 37. Four student participants were paid for participation, and others were work associates who volunteered in exchange for participation in their experiments.

### Design

**Figure 2:**
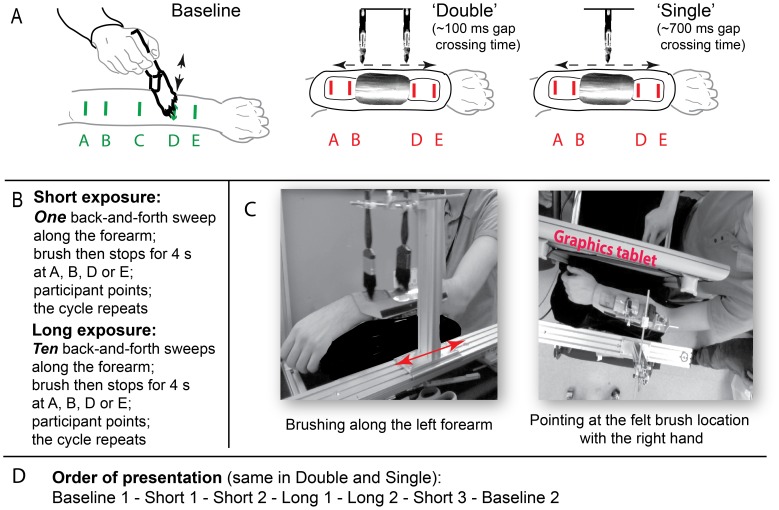
Design and procedure. A) Baseline, Double and Single experimental conditions. In Baseline, manual brushing across a bare forearm (in medio-lateral direction) provided tactile targets, and the participants pointed while the target was applied. In ‘Double’ we used the Abridging stimulus shown in Fig. 1, and in ‘Single’ a single brush moved along the same path that two different brushes covered in the Double condition. In the Double condition, one of the two brushes was always in contact with the skin, while in the Single condition, skin contact ceased while the brush was traversing the occluder. Pointing occurred when the brush paused for 4 s during its proximo-distal (or disto-proximal) motion. B) Summary descriptions of Short and Long runs, emphasizing the large difference in the duration of brushing between the stops. C) Experimental set-up. Left panel: side view of the brush carrier carrying two brushes in the Double condition. Right panel: Birds-eye view of the participant’s left, brushed arm, the graphics tablet used to record the pointing response, and the right arm executing a pointing response. D) Order of presentation of runs within each session.

We used phenomenological report and localization by pointing to determine the effects of our novel cutaneous stimulus. A repeated-measures experimental design was used and is depicted in Figure 2.

The main variable in the experiment was *traverse time* across the occluder, when the forearm was brushed longitudinally. In Double (the critical condition), traverse time was extremely short and thus the temporal continuity of skin stimulation was maintained in spite of a spatial gap in the motion path, while the other condition, Single, contained a temporal gap corresponding to the spatial gap. The Single and Double conditions were presented in separate sessions, counterbalanced across the participants.

The other variable was *exposure to brushing* before each pointing response, i.e., the number of sweeps up and down the arm before the brush stopped at the predetermined location. The number of sweeps was greater in Long than Short runs (see Fig. 2B and below for details), which were both presented within the same session in a fixed order. The order of runs was fixed rather than counterbalanced because we wanted to test for any immediate illusion (observable in Short) and any cumulative effects. In addition, the basic ability to localize tactile stimuli applied to the forearm was determined in a Baseline condition prior to the exposure to longitudinal brushing and this was repeated at the end of the session. Thus, each session comprised a series of runs (Baseline, Short, Short, Long, Long, Short, Baseline). In one session, all the Short and Long runs consisted of the Double condition, and in the other session, all consisted of the Single condition.

Detailed description of the Baseline condition and two main experimental variables, traverse time and exposure, follows. One additional variable, direction of approach, was applicable to a subset of pointing targets and is also described below.

#### Baseline

A brush was applied manually across the forearm on one of 5 target locations, A-E. Each target location was presented 6 times, in random order. The experimenter applied several back-and-forth strokes (in medio-lateral direction), and participants pointed toward the site of stimulation while the brush was applied. They usually completed the pointing response during the stimulation.

#### Traverse time

*Single (app. 700 ms traverse time)* In the Single condition, a single brush moved the whole distance between the most proximal and most distal locations (locations A and E in Fig. 2A), traversing a metal occluder, which prevented touch on the skin and provided the ‘gap’ in stimulation. The brush traversed the gap at the same speed of 14.5 cm s^−1^ as its speed before and after the gap. Travel time across the 10 cm gap was app. 700 ms. The brush stopped at one of the 4 target locations (A, B, D or E), 6 times each, in random order, and remained there for 4 s while the participant pointed at its perceived location (see Fig. 2B).

*Double (app. 100 ms traverse time)* In the Double condition, two brushes separated by 9 cm were mounted on a carrier and moved together at the same speed as in the Single condition (14.5 cm s^−1^). Only one brush was ever in contact with the skin, but – unknown to the participants - it was a different brush on two sides of the gap. Only the distal brush stimulated the distal exposed area of the skin, and only the proximal brush, the proximal area. Skin contact was maintained throughout the brushing because as soon as the distal brush moved onto the occluder, the proximal brush moved from the occluder onto the skin, and *vice versa*. Thus, in contrast to the Single condition, the temporal gap was <100 ms, much shorter than the expected value of app. 700 ms for continuous motion of the brush at uniform speed across the 10 cm occluder in its motion path. The brush stopped at one of the 4 target locations, A, B, D or E, 6 times each, in random order, and remained there for 4 s to allow the participant to point (Fig. 2B).

#### Exposure

*Short (1–2 up-and-down brush sweeps)* The brush made 1 to 2 elbow-to-wrist-to-elbow sweeps (lasting app. 1–3 s in the Double condition and 3–6 s in the Single condition) between 4-s stops during which the participant pointed at its felt location. A total of 24 pointing trials were performed, 6 for each target.

*Long (10 up-and-down brush sweeps)* Long runs began with 4 min (Double) or 7.5 min (Single) of non-stop brushing to which the participants were asked to attend. During this period, they were also reminded that at the end of the experimental session, they would be asked to report *where* they had felt the brushing. Following this period, brushing was interrupted with 4-s stops at locations A, B, D or E, during which the participant pointed at the felt location of the brush. Brushing between the 4-s stops consisted of ten elbow-to-wrist-to-elbow sweeps, lasting on average app. 15 s in the Double condition and 28 s in Single. A total of 24 pointing trials were performed, 6 for each target.

#### Direction of approach

This variable was manipulated within Short and Long runs, and for target locations B and D only. In those conditions, a brush travelled across the occluder (as explained above, in the Double condition, there were in fact two brushes, in which case one mounted onto the occluder, while the other descended on the opposite side). In the pointing trials, the brush could approach target locations B and D from across the occluder (Bridging approach), or from elbow/wrist (Non-bridging approach) before stopping to initiate the pointing response. Each approach to targets B and D occurred 3 times, in random order. Locations A and E could only be approached from across the gap. We controlled direction of approach because the across-the-occluder approach could potentially result in greater mislocalization than the approach from the opposite direction.

The dependent variable in all conditions was the location of the open-loop pointing response to one of the 4 target positions (5 positions in the Baseline condition). Two target positions were near the edges of the occluder (targets B and D), and two were near the wrist and elbow (A and E). The additional target (C) used in the Baseline condition was in the middle of the forearm and hidden by the occluder in all other conditions. Its purpose was to suggest to the participants that stimulation could also occur in the middle of the forearm.

Phenomenological report was the second dependent variable, obtained once after each of the two sessions (Single and Double).

### Apparatus and Set-up

A computer-controlled brush carrier (WS70, Excitron Corporation, Colorado, USA; see Fig. 2C) executed the motion sequence. One or two brushes, 1 cm thick and 4 cm wide, were mounted on the carrier and their height was individually adjusted to individual participant’s left forearm to ensure good contact with the skin. Their wide side was orthogonal to the motion path. Graphics tablet (Wacom XD-1218-U 12×18″) and MousePro freeware were used to record pointing responses. The tablet was mounted vertically between the participant’s two arms. The apparatus made noise during carrier motion, which the participants could hear. Both in the Single and Double conditions, the noise suggested continuous motion.

Blindfolded participants sat comfortably with their left shoulder close to the graphics tablet (Fig. 2C, right panel). The forearm was placed on a rest consisting of two narrow (2×7 cm) cylinders wrapped in fabric, one under the base of the palm, the other near the elbow. This position was maintained throughout the experiment, unless the participant asked to move the hand because it felt uncomfortable or was beginning to feel numb. In such – rare – cases, the experimenter encouraged them to raise the forearm and move the hand. The right hand was resting on the table approximately 30 cm away from the graphics tablet, holding a stylus.

Separation between two proximal targets, A and B, was app. 3.6 cm, with the same separation for distal targets D and E. Targets B and D were app. 12.0 cm apart, and A and E app. 19.2 cm apart. Lines drawn on the skin indicated locations for the experimenter to brush in the Baseline condition. A custom program (Spike2 v. 6 software, Cambridge Electronic Design, Cambridge, UK) was used to drive the carrier to the locations in the Double and Single conditions.

### Procedure

Participants were familiarized with the procedures by performing one Baseline and one Short run. In most cases, these immediately preceded the experiment. All brushing was applied to the left forearm. The task of a blindfolded participant was to point with the right arm at the felt location of the brush. In the Baseline condition, the pointing occurred during manual medio-lateral brushing, and in the other conditions, after the brush stopped during its proximo-distal motion. Pointing was recorded via a graphics tablet and the participant received no feedback. Participants were not informed about the details of the set up – that they were brushed with two brushes in the Double condition, or about the occluder placed on their forearm. They knew that ‘some kind of sleeve’ was there. After the experiment, they were given a drawing representing the forearm, and asked to indicate where they felt the brushing throughout the session. They were also to indicate on the drawing how intense the brushing felt in different locations, and to comment on any other aspects of the experience.

Double and Single experimental conditions were presented in separate blocks, on separate days, with an order counterbalanced across participants. Double sessions lasted approximately 1.5 hours, and Single 2 hours, including practice. Each session consisted of 9 runs, separated by a few minutes. The runs presented within Double and Single sessions followed the same sequence: Baseline practice, Short practice, Baseline 1, Short 1, Short 2, Long 1, Long 2, Short 3, Baseline 2 (Fig. 2D).

Approximate durations were 3 min for Baseline, 2 min for Double-Short, 3 min for Single-Short, 12 min for Double-Long and 20 min for Single-Long. All runs had 24 pointing trials, except for Baseline, which had 30. Pointing targets were presented in random order, with the constraints that all the targets were presented twice before any of them was presented again, and that the two presentations of B and D within any two-presentation cycle had different directions of approach.

### Data Analysis

Raw data were the locations of the pointing responses along a single dimension. Responses to targets A, B, C, D and E were checked for outliers and excluded if they deviated by more than 2.5 SD from the average SD computed across all participants for all targets in a given run (e.g. Short 1). No more than two (out of 6) data points per participant per target were to be excluded. Approximately 2% of outliers were excluded on this basis, with relatively even spread across experimental conditions.

Distances between responses to targets B and D, and between responses to targets A and E were calculated for each run for each participant and were subtracted from the corresponding values in Baseline 1 in the same session. Short 1 and Short 2 runs were pooled, and so were Long 1 and Long 2 runs. Short 3 data were missing (due to experimenter error in recording the pointing responses) for two participants, one in the Single and one in Double condition. These participants’ data were otherwise complete, and the missing Short 3 data were replaced with estimates based on the group mean for that condition, corrected by the difference between the participant’s overall mean and the grand mean. Repeated measures ANOVAs (using type III sum of squares) conducted in SPSS were used to test for the effect of traverse time, by comparing Single and Double conditions, and the effect of exposure by testing for trend (using polynomial contrasts in SPSS) across successive runs within a single session (Short 1, 2; Long 1, 2; Short 3; Baseline 2). Two directions of approach to targets B and D were compared using the paired samples t-test.

## Results

### Phenomenological Reports

**Figure 3:**
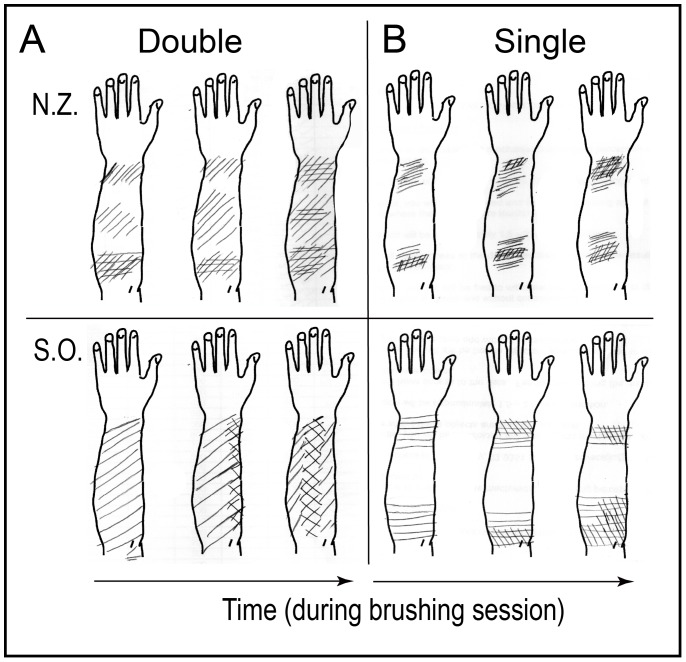
Representative drawings of two participants indicating the areas where they felt the brushing. More heavily shaded areas represent more intense brushing sensation. Note how percepts differed in A) Double versus B) Single, and how they changed over time (see text for details).

Phenomenological reports – verbal and drawings – show that in the critical condition, Double, each of our 10 participants perceived continuous motion all along the forearm, 8 of them most of the time, and 2 at least some of the time (one felt a gap but it then closed, and the other felt that his own forearm had ‘a dip which was not being brushed’). When shown the 10-cm wide occluder on their forearm afterward, participants were often surprised. Representative drawings for this condition are shown in Figure 3A; note that they have no gap in the place where one would be expected given the presence of the metal occluder.

By contrast, in the Single condition, most participants, including N.Z. and S.O. (Fig. 3B) felt a large gap. Only one person reported no gap at all, and three stopped feeling the gap toward the end of the session.

The drawings illustrate also how percepts changed over time: in the Double condition, the perceived intensity of stimulation gradually increased in the area that received no physical input, and small perceived discontinuities closed. In the Single condition, the large initial gap gradually decreased in size either by areas getting closer to each other (N.Z.) or increasing in size (S.O.). These changes were described by 9 participants. Thus it appears that completeness of perceptual completion increased over time, and that even in the Single condition there was a tendency to complete the motion although in most cases it did not close the gap.

### Pointing Results

The pointing responses indicated that the perceived positions of the brush touching the skin on opposite sides of the gap were heavily biased towards the middle of the forearm.

**Figure 4:**
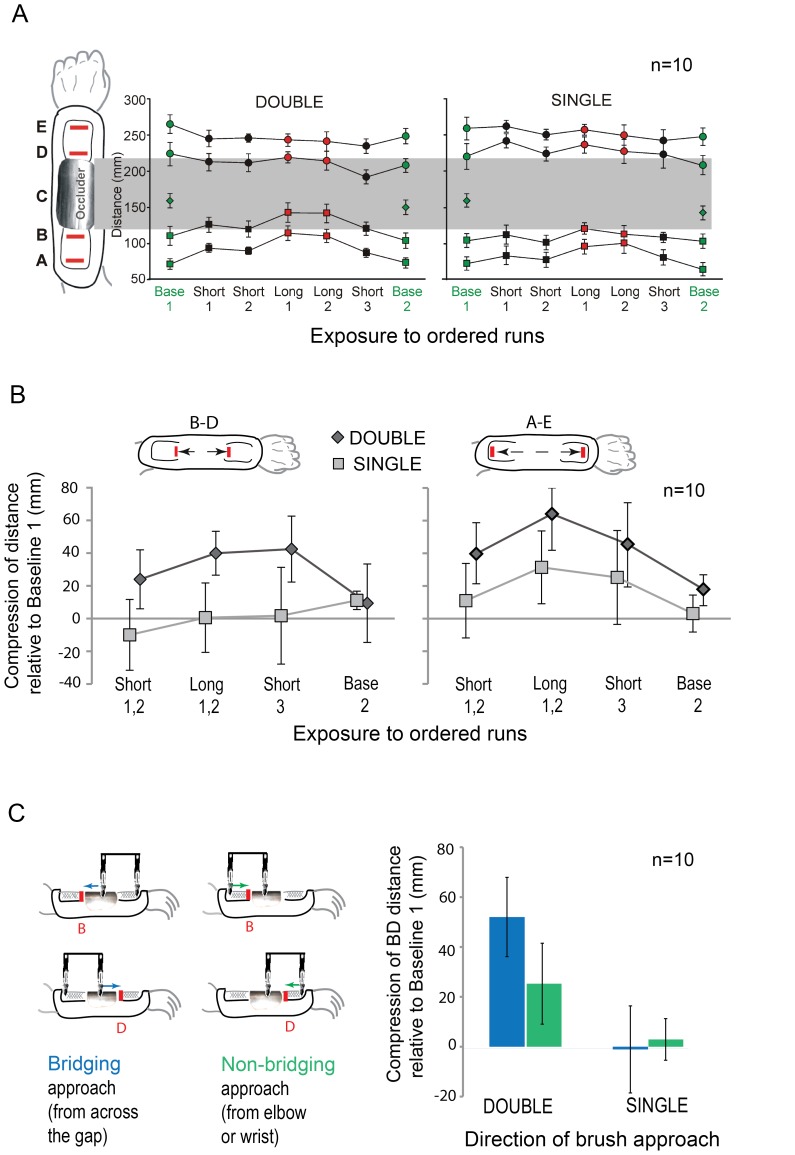
Results of the pointing task. A) Mean localization responses to targets A, B, C, D and E for each run in each session. Error bars represent 95% CIs for within-subject designs [19]. Targets are indicated on the scaled illustration of the forearm. The grey box across both panels represents the location of the occluder, which was removed from the arm during each Baseline run (Base 1 and Base 2, green symbols). Lines connect responses to the same target location at different moments in time. Order of runs along the x axis is the same as their order of presentation in the experiment. Note a large pointing bias towards the middle of the forearm during the Short (black symbols) and Long (red symbols) runs in the Double condition (left panel). B) Distance between mean responses to the external targets (A and E) and internal targets (B and D) expressed as a deviation from Baseline 1. Positive values indicate compressive mislocalization. Note compressive mislocalization is present in the Double condition (diamonds) but not Single (squares) for BD (left panel), and is much greater in the Double than Single condition for AE (right panel). C) Left panel of the figure illustrates two directions of brush approach (Bridging and Non-bridging) for targets B and D. Right panel shows the distance between responses to targets B and D in the combined Long runs (Long 1,2) as a function of direction of brush approach. Distances are expressed relative to Baseline 1 (as above). Note that both brush approaches in the Double condition result in compressive mislocalization, but that it is greater with the Bridging (blue) than the Non-bridging (green) approach; the Single condition shows no bias for either direction.

Detailed group results - responses to each target location in every experimental run in the order in which they were presented - are shown in Figure 4A. Each line connects responses toward one target location in different runs throughout the experimental session. An unchanging response would result in a straight, horizontal line. The responses in the critical, Double condition (left panel) show large deviations from Baseline 1 (green data points on the left). The responses in Short and Long runs on both sides of the gap were affected by brushing, with points on both sides of the occluder (grey box) shifting closer to the middle part of the forearm. Responses during Baseline 2 (green symbols on the right) are similar in both sessions. In contrast, the responses in the Single condition (right panel) show little change in perceived location over the course of the session, especially for the targets next to the occluder (B and D). Responses to the two external targets (A and E) are shifted toward the middle of the forearm although not as much as in the Double condition.

To summarize the results, distances between the mean responses to the external targets (A and E) and between the mean responses to internal targets (B and D), were subtracted from the corresponding distances in Baseline 1. In Baseline 1, the mean BD response distance was 114 mm in Double and 115 mm in Single, and the respective AE distances were 189 and 191 mm (physical distances were 120 mm for BD and 192 mm for AE). The results are presented in Figure 4B, with positive values indicating the compression of distance.

The critical variable, *traverse time*, had a large effect on both sets of targets: compressive mislocalization is greater in the Double than Single condition (see Fig. 4B, both panels). In the Long runs, in which the exposure to brushing prior to the pointing response was the greatest, the compression of BD distance in the Double condition was 38.6 mm (or 34%) relative to Baseline 1, in comparison to only 0.9 mm (or 0.8%) compression in the Single condition. The compression of AE distance in the Double condition was 63.8 mm (or 33%) relative to Baseline 1, in comparison to 31.8 mm (or 16.8%) compression in Single. In the Single condition, responses to BD changed little throughout the brushing sessions, but the AE responses shifted towards the middle of the forearm. *Exposure* also affected the responses: compressive mislocalization increased with exposure (BD in the Double condition, and AE both in Double and Single) but had almost completely disappeared during the final run in each session when targets were presented by manual brushing across the forearm (Baseline 2).

Statistical analyses support the above observations. Two separate 2×4 ANOVAs were conducted, one for BD and one for AE, with factors traverse time (Double, Single) and exposure (Short 1,2; Long 1,2; Short 3; Baseline 2). Responses to targets BD show significant main effects of traverse time (F(1,9) = 7.19; p = .025) and exposure (F(1.5, 13.5) = 3.03, p = .047, with Greenhouse-Geisser correction), as well as a significant interaction (F(2.4,21.5) = 5.08, p = .012, with Greenhouse-Geisser correction). As the left panel of Figure 4B suggests, the significant interaction is due to a highly significant quadratic trend in the Double condition (F(1,9) = 26.26, p = .001), and not in the Single condition (F(1,9) = .007, p = .937). Linear trend for Single was also not significant (F(1,9) = 4.32, p = .07).

Responses to targets. AE also show significant main effects of traverse time (F(1,9) = 6.02, p = .037) and exposure (F(3, 27) = 12.05, p = .0001), but no interaction (F(3,27) = 1.04, p = .392). The exposure exhibited a significant quadratic trend (F(1,9) = 33.22, p<.0001). As the right panel of Figure 4B and the absence of interaction show, a quadratic trend is present in both Double and Single conditions. Thus unlike BD responses, AE responses in the Single condition were influenced by brushing although to a lesser extent than in the Double condition.

*Direction of brush approach* to the target position was also of interest (see Methods for details). The two directions were Bridging (from across the occluder to either target B or D), and Non-bridging (directly from the elbow to target B, or from the wrist to target D). Results obtained in Long 1,2 are shown in Figure 4C (right panel) as compression of response distance relative to Baseline 1, with larger values representing greater compressive mislocalization towards the middle of the forearm. A 2×2 ANOVA with factors traverse time and direction of approach shows a main effect of traverse time: both directions of approach resulted in greater compressive mislocalization in the Double condition than Single (F(1,9) = 12.00, p = .007). The main effect of direction of approach was also significant (F(1,9) = 5.09, p = .05), as well as the interaction between the two factors (F(1,9) = 7.76, p = .021). The Bridging approach had a greater effect (52.0 mm compression or approximately half the width of the 10-cm occluder) than Non-bridging approach (25.2 mm compression) in the Double condition (t(9) = 2.93, p = .017). Nevertheless, the Non-bridging approach also created a significant compression (its 95% CI is 5.3–45.1 mm). Thus, as well as observing shifts in perceived position when the two sides of the gap were stimulated in sequence (46% reduction in gap size relative to Baseline 1), we also found a significant bias (22% reduction in gap size relative to Baseline 1) when the brush approached the skin areas next to the gap from the direction of the wrist or elbow. Both effects were close to zero in the Single condition.

Bridging and Non-bridging approaches resulted in a similar tendency in Short 1,2, with 34.3 mm (95% CI: ±15.11) and 20.4 mm (95% CI: ±23.79) compression, respectively, but this difference was not statistically significant (t(9) = 1.18, p = .267).

## Discussion

We describe a new form of perceptual completion, i.e., a sensation of continuous motion along the skin despite a very large (10 cm) gap in stimulation in the middle of the motion path. We also describe a gross mislocalization of touch on the skin on either side of the gap, an effect we term ***‘abridging’*** because the reconstructed overall motion path is much shorter than the path obtained in the baseline condition. The comparison between the critical condition (Double) and a control (Single) shows that the abridging was not a consequence of the spatial gap in stimulation itself but of the traverse time. Both conditions had a gap, but in the Double condition, in which we see the abridging, the gap traverse time was <100 ms, compared to the 700 ms in Single.

While some spatial compression occurred in the Single condition, it was a consequence of mislocalization of the external targets (Fig. 4B, right panel) rather than those next to the gap, which show no such trend (Fig. 4B, left panel). By contrast, mislocalization in the Double condition was most pronounced for targets next to the gap, strongly suggesting that the reason for mislocalization is different in the two conditions. We speculate that in the Single condition, it reflects a tendency to point toward the centre of the brushed area on each side of the occluder.

The abridging effect brings to mind sensory saltation because both are compressive mislocalizations (i.e., perceptual length contractions, the term introduced by Goldreich [20]) induced by short temporal intervals between successive stimulations. The tactile saltation illusion or cutaneous rabbit is a bias in perceived location of a discrete tap or vibrotactile stimulus towards a stimulus delivered shortly after or before [11]–[15]. It is usually induced by stimulating two or more positions on the forearm at temporal intervals from around 60 ms to 200 ms (for example, two quick taps in one location followed by another at a different location, result in the sensation that the middle tap is spatially displaced towards the last). These stimulus conditions differ markedly from ours, because our motion stimulus is continuous, and it moves at a constant speed before and after crossing the gap. The similarity is in the temporal separation of <100 ms for brush stimulation on two sides of our 10-cm gap, which is comparable to the temporal intervals between the taps in the saltation paradigm.

Illusory spatial compression also occurs with continuous motion stimuli (rather than just discrete taps used in the sensory saltation), when they move at the speeds comparable to our across-the-gap speed of approximately 100 cm s^−1^. For example, a 4 cm spatial interval is perceived as half as long when brushed at 100 cm s^−1^ compared to 15 cm s^−1^ (estimated from Fig. 2, [21]). It is not certain that this effect can be attributed to speed alone because the speed was confounded with the temporal interval required to complete the movement. Also, the stimulus differs from ours because we did not brush across the gap. Nevertheless, the spatiotemporal parameters resulting in the spatial distortion are remarkably similar to those used in the sensory saltation and our own paradigm, suggesting that these distortions could have a common basis.

An important difference between our results and the distortions described above is that we also found a significant bias when the brush approached the skin areas next to the gap (targets B and D) from the direction of the wrist or elbow (Non-bridging direction of approach, see Fig. 4C). When approaching from that direction, the brush moved at 15 cm s^−1^, not 100 cm s^−1^; the 100 cm s^−1^ across-the-gap motion occurred app. 0.5 s prior. It also did not fit stimulus conditions for saltation because there was no spatially remote stimulus immediately prior or after stimulation of the target position. Another difference is that the compressive mislocalization occurred not only in skin areas adjacent to the gap (targets B and D), but also in other areas (targets A and E). This was likely the consequence of our use of the continuous motion stimulus, which covered a 3.6 cm distance before and after each ‘leap’ across the gap. We propose that motion defines relative locations of skin patches in its trail, including those on the continuous path. Therefore, when the borders of the gap were drawn together due to the short across-the-gap time, so was the surrounding skin. We did not test skin areas beyond the brushed zone but predict that the effect, if there, would be smaller than on the skin covered by the brushing. Mislocalization of targets A and E was also found in the Single condition, but it was significantly smaller in size and likely caused by a different factor, which possibly also increased the bias for targets A and E in the Double condition over and beyond that caused by the brief gap traverse time.

These findings, combined with the finding that a longer duration of brushing results in a greater bias (Fig. 4B), suggest that at least two processes are involved - one resulting in an immediate position illusion, and one creating a longer lasting, cumulative bias that spreads to adjacent skin areas. This cumulative effect seems to be specific to the stimulus that provoked it: there is no evidence that it distorts responses to other tactile stimuli, such as motion in a direction orthogonal to the brushing motion (Baseline 2 was similar in Double and Single, Fig. 4B).

### Why did Abridging Occur?

**Figure 5:**
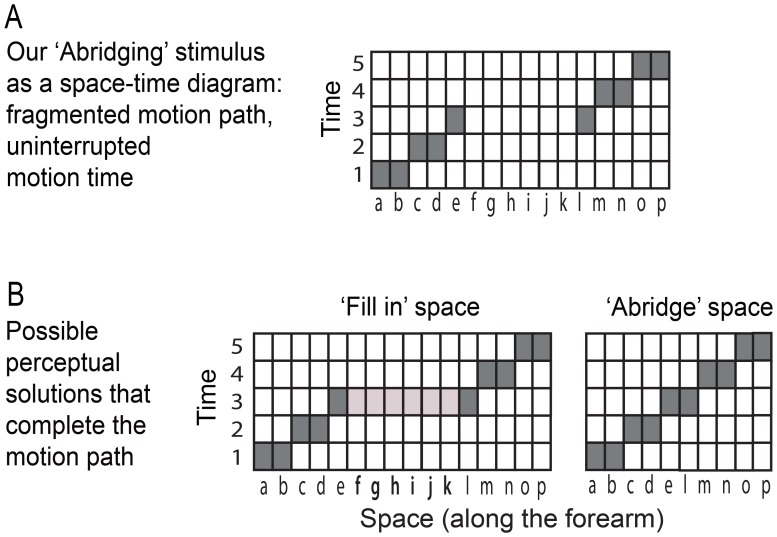
Abridging stimulus and its possible perceptual solutions as space-time diagrams. A) The stimulus. B) Two perceptual solutions, ‘Filling in’ and ‘Abridging’; note that they both complete the motion path. Filling in does not require a modification of the spatial dimension while abridging requires that it is shortened.

We propose that abridging had a specific role, which is to achieve perceptual completion of the spatial gap in stimulation *and* to make the spatial and temporal aspects of the event congruous. Consider first the completion. As Figure 5A shows, the motion path was incomplete i.e., fragmented. Fragmentation of input is common in everyday perception and, as richly documented in vision research [22], the sensory system is well equipped to overcome it and achieve an uninterrupted perception of objects and events. One way to achieve completion is by ‘filling in’ of a non-stimulated area with sensory attributes from the surround, as happens with the blind spot [23]. Completion of a motion path could in principle be achieved by ‘filling in’ the physical gap in stimulation with sensation of motion from the surround (Fig. 5B, left), or it could be achieved by ‘abridging’ the motion path - bringing together in a hypothetical map the patches of skin bordering the opposite sides of the gap (Fig. 5B, right). Here, abridging occurred in the Double condition. It might seem like a costly solution, given that it involves a distortion of the spatial dimension. Why did it occur, if filling-in could achieve completion? The answer is in its second proposed role: abridging resolves the discrepancy between space and time present in our stimulus. It accounts for temporal continuity (in the presence of spatial discontinuity), which filling-in does not.

Phenomenological reports show that to some extent, completion was developing in the Single condition (although the gap in most cases did not disappear) but in the absence of clear evidence of abridging for skin areas next to the gap (targets B and D, see Fig. 4). This is consistent with the above analysis and interpretation: filling in, rather than abridging, would be sufficient to close the spatial gap when spatial and temporal aspects of the stimulus are congruent, as they were in the Single condition.

Filling in likely also contributed to some extent to the closing of the gap in the Double condition. Some participants reported that while the motion sensation extended all the way through the forearm, ‘prickliness’ of the brush was not felt in the middle. This is similar to other filling-in phenomena, where the non-stimulated area is filled with some, but not necessarily all stimulus features present in the surround [22]. On the other hand, it is not consistent with abridging: the fully abridged motion path should, by definition, exclude the non-stimulated area and the reported sensations should all originate in the stimulated skin.

To summarize our proposed relationship between completion and abridging, we consider the fragmentation of input (that calls for spatial completion) a necessary, but not sufficient, condition for abridging. It is not a sufficient condition because fragmentation can also be overcome by filling in, but it is a necessary condition because abridging is no more than a way to overcome fragmentation. Thus we predict that a modification in the stimulus pattern such that it no longer requires completion would eliminate abridging. Abridging may also be absent from a stimulus that requires completion, as long as it does not contain space-time discrepancies similar to that we created here. An example would be motion that does not ‘leap’ across the spatial gap but occurs on each side of it by brushing across the forearm, i.e., in the direction orthogonal to its long axis. In that case, even though the input is fragmented (the middle of the forearm lacks stimulation), potentially resulting in completion, there should be no abridging.

### The Role for Gestalt in Defining Maps

The implicit notion in the above discussion of fragmentation of input is that there is some constraint that does not allow our participants to have a veridical percept of the stimulus pattern in the Double condition. A veridical percept would be that two separate brushes are moving across the skin. Rather, the participants perceived a single moving object, which resulted in closure of the gap and abridging. The cues suggesting that there was only one object were a uniform speed, continuing motion in the same direction on both sides of the gap, and the characteristics of the brush such as its stiffness.

Gestalt psychologists [17] have provided the theoretical framework and insights into the laws of grouping of elements into meaningful wholes, which help the sensory system organize input and perceive objects and events. Most studies of Gestalt laws have been concerned with vision, but the same principles apply in other senses, including touch [24]. These laws can be seen as heuristic cues for the physical objects, “in that a segment of the {(…) sensory} field that belongs together perceptually has a counterpart in a portion of the outer world that ‘hangs’ together physically” (p. 16, [18]). We think that this is the best framework for interpretation of our results and that gestalt principles are super-ordinate drivers for definition of maps and plastic changes that would trump any local triggers, such as co-stimulation of skin areas, if in conflict with them.

### Do Motion Mechanisms Underlie Abridging?

Our stimulus involves coactivation of the areas on the opposite sides of the gap, and thus builds upon an idea pioneered by Merzenich and colleagues, that coactivation of neurons by co-stimulation of skin loci results in these areas being represented together in cortical maps. However, we use a motion stimulus, rather than coactivation alone. Whether motion is crucial in the present context needs to be further investigated, but we propose that it is.

Motion stimuli provide co-stimulation, but also sequential stimulation, both of which are subsumed under the more general map-building principle of spatiotemporal proximity [25], [26]. That motion is crucial is a long held idea [16], which, in its modern form [27], suggests that by creating correlated activity, motion strengthens relationships between neurons representing adjacent localities, and that these neighborhood relations are the essence of the position signal (also known as local sign) that each neuron presents to consciousness.

This emphasis on motion may seem unjustified given that timing-dependent compression of perceived space is also observed in the tau effect described in the introduction. Unlike abridging and saltation, stimulus conditions for tau do not necessarily result in perception of motion (apparent movement was considered “the complicating factor” by the authors of the original work [10], p. 203). Thus motion mechanisms are not the only cause for time-dependent spatial distortions, even if they are, as we propose, involved in the abridging.

A parsimonious explanation for all three effects - tau, saltation and abridging – would involve a shared cause. Therefore, if perceived motion is not a necessary condition for one of them, that would suggest that cause for all three should be sought elsewhere. However, statistical regularities of *physical* motion – not necessarily resulting in perceived motion - could be the explanatory principle underpinning all three effects. Stimulation of different areas on the skin occurs due to movement of our limbs, for example when we grasp objects, or brush against them, or due to object motion against our body. There are regularities in those types of stimulation, and one of them could likely be summarized as follows: ‘the shorter the time between stimulation of point A and point B on the skin, the shorter the distance between them’, or simply that short inter-stimulus intervals mean that points of stimulation are close. A similar explanation, a Bayesian ‘low speed’ prior, has been proposed for the saltation and tau effects [28].

Nevertheless, if the spatiotemporal window were tight enough for the stimulus to trigger the motion percept, we would also experience motion as we do in abridging and saltation. In that case, the motion sensitive neurons located in the primary somatosensory cortex, in particular areas 3b, 1 and 2 [29], are engaged and represent the most likely neural substrate involved. We propose that the functional connections related to the position signal between the lower-level somatosensory neurons are continuously updated based on the feedback from the motion neurons. This top-down influence shapes the local sign (i.e., perceived position arising from the activity in a particular neuron) of sensory neurons to be consistent with a plausible perceptual solution given the current stimulation pattern (context). Recurrent interactions between sensory neurons at different levels have been proposed to underlie boundary finding in vision utilizing a larger spatial context (see Figure 8-2 in [30]). It has also been proposed that such interactions may result in longer lasting neural changes.

### Plasticity and Specificity of the Abridging Effect

Is the Abridging effect a precursor to a more lasting change in the receptive fields and ‘local signs’ of sensory neurons? Consistent with what we said above, we think that it is. It fulfills the need to perceive continuous objects and events in spite of discontinuities in the patterns of input that impinge on our senses. Although our stimulus represents a temporary, external cause of discontinuity in the sensory input, it resembles internal, lasting causes because the same patch of skin is repeatedly skipped – as if it cannot register input. In this way we simulate the physical loss of an area from the receptor surface, where the remaining skin is surgically sutured together, resulting in new neighborhood relations between previously remote receptors. Discontinuities caused by lasting internal causes create the need for lasting, plastic change of the sensory apparatus. In this view, completion is only the first phase of a more lasting plastic change that will occur if the conditions of stimulation last [31], and cortical plasticity studies [7] show that such plastic changes occur in sensory neurons. Cholewiak [13] expressed a similar view with regards to the tau effect and saltation, which, he proposes, “indicate a built-in propensity at some level in the nervous system for complex interactions (…) inborn and emergent like the Gestalt principles of perceptual organization. However, the single-unit physiological data cited above may indicate that, through related experiences, the physical connections and interactions can be strengthened.” (p. 871).

Specificity of the effect depends on the types of neurons involved in its creation. Since the Abridging stimulus engages neurons sensitive to direction of motion along the forearm, it is possible that mislocalization only occurs for the stimuli moving along the same path, and not for stationary stimuli, or stimuli moving in orthogonal direction. On the other hand, if the effect results in redefinition of relative position in the neurons specifying position rather than motion, it could generalize to other kinds of stimuli. We currently have no evidence for the latter possibility - we observed no bias in the post-test (Baseline 2), in which we used motion across the arm to indicate target locations. This suggests that the ‘map’ distorted by our stimulation is a stimulus-specific map.

## Conclusion

Our stimulus produced a novel cutaneous illusion that resulted in gross mislocalization of touch. The Abridging illusion suggests that sequential activation as part of a motion signal defines neighborhood relations amongst cutaneous afferents. The illusion we describe may share mechanisms with sensory saltation and the tau effect, which rely on discrete stimuli, as well as the illusory compression of space observed with high-speed continuous motion. However, we show that the mislocalization persists beyond the high-speed motion event to affect stimuli moving at low speed, extends beyond the skin parts directly involved in high-speed motion, and increases with exposure. We argue that the overall stimulation pattern, not shared with saltation or tau, is largely responsible for the effect. The lasting and cumulative effect suggests adaptation of a dynamic, stimulus-specific map of the skin surface.

The abridging illusion taps into a low-level representation of the skin surface rather than the high-level multimodal body representation that underlies some well-known illusions (e.g., the rubber hand illusion). Thus it may provide a tool to probe how a low-level single modality representation influences body perception and action.
